# Medical Society of the County of Erie

**Published:** 1907-06

**Authors:** 


### SOCIETY PROCEEDINGS.

### Medical Society of the County of Erie.

THE regular quarterly meeting of the Medical Society of the County of Erie was called to order at 8:45 o'clock p.m., on Monday, April 8th, 1907, in the rooms of the Buffalo Society of Natural Sciences, by the president, Dr. A. H. Briggs.

Minutes of the regular meeting held January 14, 1907, and of the special meeting held February 19, 1907, were read and on motion approved, as were also the minutes of council held April 1, 1907.

Dr. Grosvenor, of the auditing committee, presented the following report:

*To the Medical Society of the County of Erie*:

Your committee appointed at the last regular meeting of the society, January 14, 1907, to audit the accounts of the treasurer for the year 1906 has performed that service and has found as follows:

| | |
|---|---|
| Balance on hand, January 1st, 1906............ | $ 93.91 |
| Total receipts during the year 1906............. | 1,175.25 |
| Total balance and receipts...................... | 1,269.16 |
| Total disbursements during 1906.............. | $1,083.72 |
| Balance on hand January 1st, 1907............ | 185.44 |

These figures are in accord with the report of the treasurer made at the last regular meeting of the society.

The treasurer holds vouchers for all disbursements.

The balance on his books January 1st, 1907, agrees with the balance in bank to his credit as treasurer, as shown on his bank book.

We therefore certify that the accounts of Dewitt C. Greene, treasurer of the Medical Society of the County of Erie for the year 1906, are correct.

On January 1st, 1907, 175 members were in arrears for dues, to the amount of $1,410.00. This amount does not include members who have left the county under indebtedness to the society, nor does it include members who died before liquidating the dues which they owed to the society.

Your committee recommends that proper and effective measures be adopted for collecting all collectible dues which have accrued against members of the society.

A copy of the report of the treasurer for the year ending Dec. 31, 1906, and a list of members who were in arrears for Dues on January 14, 1907, accompany this report of the auditing committee.

J. W. GROSVENOR
GEORGE L. BROWN
DELANCEY ROCHESTER
Auditing Committee.

Buffalo, N. Y.
April 8, 1907.

Copy of report of Dr. Dewitt C. Greene, treasurer of the Medical Society of the County of Erie, for the year ending Dec. 31, 1906:

RESOURCES.

| | | | |
|---|---|---|---|
| Jan. | 1. | Balance on hand as per report for 1905...... | $ 93.91 |
| | 8. | Louisa Busch, fine......................... | 250.00 |
| | | Dues collected, 1906, per cash book........ | 925.25 |
| | | Total resources.... | $1.269.16 |

1906.

LIABILITIES.

| | | | |
|---|---|---|---|
| Jan. | 6. | Secretary, expense account | $ 8.00 |
| | 8. | John Lord O'Brien, legal services, 1905 | 250.00 |
| | 23. | Dr. Edwd. Clark, expense to Albany | 20.00 |
| | 23. | Red Jacket Press, printing for secretary | 2.50 |
| | 29. | Med. Soc. State New York, Transactions 1904-5 | 25.00 |
| Mar. | 1. | John Lord O'Brian, legal services | 50.00 |
| | 6. | Secretary, expense account | 12.00 |
| Apr. | 13. | C. W. Miller, janitor | 3.00 |
| May | 9. | Rubber stamp for treasurer | .75 |
| | 10. | Postage stamps for treasurer | 6.00 |
| | 26. | Red Jacket Press, printing for secretary | 18.75 |
| | 26. | Red Jacket Press, printing for secretary | 3.00 |
| | 26. | Secretary, expense account | 24.78 |
| | 26. | Postage stamps for treasurer | 2.00 |
| June | 6. | R. L. Polk, Medical Register | 6.00 |
| | 12. | Truckman, conveying transactions to meeting | .75 |
| | 29. | N.A.T. Carrell, printing | 13.20 |
| Dec. | 13. | Buff.Med.Journal to August 1907 | 2.00 |
| | 13. | Postage stamps for treasurer | 2.00 |
| | 29. | Secretary, expense account | 28.89 |
| | 31. | Red Jacket Press, printing for secretary | 14.10 |
| 1906. | | Paid State Treasurer, dues | 591.00 |
| | | Total disbursements | $1,083.72 |
| | | Balance on hand January 1st, 1907 | 185.44 |

DEWITT C. GREENE,
Treasurer.

Dr. Krauss moved to receive, accept, and place the report on file. Carried.

The secretary wanted to know if the list of members submitted as in arrears should be published. He was informed that this was not the intention; the list was merely for the information of the society.

On motion of Dr. Kennerson, the auditing committee's report was referred to the membership committee which is still at work on the subject of revising the membership list.

The president submitted the following communication:

DR. A. H. BRIGGS,

President of the Medical Society of the County of Erie.

Dear Sir: At the regular March meeting of the staff of the Erie County Hospital the following resolution was passed—namely, that the recommendations of the tuberculosis committee of the Charity Organisation Society of Buffalo concerning changes at the tuberculosis ward of the Erie County Hospital, together with the proposition for a separation of the Erie County Hospital from the Erie County Almhouse, be called to the attention of the officers of the Medical Society of the County of Erie, with the request that a special meeting of that society be called to consider the matter, and that the members of the Erie County Homeopathic Society be invited to be present and take part in the meeting.

Pursuant to this resolution, I would respectfully request that such a meeting be called.

MARSHALL CLINTON,
Secretary.

Moved by Dr. Kennerson that this matter be considered at the next regular meting of this society.

Dr. Bennett hoped this motion would not prevail, as it would delay an important matter too long, and he also expressed the desire that the Homeopathic society be invited to be present. He moved, in amendment, that it be considered at a special meeting, the date to be fixed by the Council.

The amendment was lost and the motion was then carried.

No candidates were proposed and no report was received from the committee on membership. No further business coming up for consideration, the president then announced the literary part of the program, and introduced Dr. Willliam C. Krauss, who read a paper on the necessity of esablishing a bacteriological laboratory in every community.

Dr. Grosvenor stated that the facts presented in Dr. Krauss's paper were certainly remarkable, and suggested that similar papers be presented at every county society in the state, for the purpose of agitation along this line.

The next essayist was Dr. Vertner Kennerson who presented a report of nine gunshot wounds of the body.

Dr. George E. Fell gave a short talk on his recent observations in the western states, after which the society adjourned.

FRANKLIN C. GRAM, M.D.
Secretary.

MEETING OF THE COUNCIL.

A meeting of the Council of the Medical Society of the County of Erie was held at the University Club, Buffalo, N. Y., Monday, April 1, 1907, at 5 o'clock p.m.

Present—the president A. H. Briggs, second vice-president James S. Smith, and secretary F. C. Gram.

It was decided to hold the regular April meeting on Monday, April 8, 1907, at 8:30 o'clock p.m., at the rooms of the Society of Natural Sciences, providing the secretary can secure the use of the rooms.

The program for the meeting was also arranged.

The Council then adjourned.

Franklin C. Gram, M.D.
Secretary.

